# PD-L1 and HIF-2α Upregulation in Head and Neck Paragangliomas after Embolization

**DOI:** 10.3390/cancers15215199

**Published:** 2023-10-29

**Authors:** Alessa Fischer, Umberto Maccio, Katharina Wang, Juliane Friemel, Martina A. Broglie Daeppen, Diana Vetter, Kuno Lehmann, Astrid Reul, Mercedes Robledo, Constanze Hantel, Nicole Bechmann, Karel Pacak, Kathrin Zitzmann, Christoph J. Auernhammer, Ashley B. Grossman, Felix Beuschlein, Svenja Nölting

**Affiliations:** 1 Department of Endocrinology, Diabetology and Clinical Nutrition, University Hospital Zurich (USZ), University of Zurich (UZH), Rämistrasse 100, CH-8091 Zurich, Switzerland; 2 Department of Pathology and Molecular Pathology, University Hospital Zurich, CH-8091 Zurich, Switzerland; 3 Department of Medicine IV, LMU University Hospital, LMU Munich, 80336 Munich, Germany; 4 Department of Otorhinolaryngology, Head and Neck Surgery, University Hospital, CH-8091 Zurich, Switzerland; 5 Department of Visceral and Transplantation Surgery, University Hospital, CH-8091 Zurich, Switzerland; 6 Hereditary Endocrine Cancer Group, Spanish National Cancer Research Center (CNIO), 28029 Madrid, Spain; 7 Centro de Investigación Biomédica en Red de Enfermedades Raras (CIBERER), 28029 Madrid, Spain; 8 Medizinische Klinik und Poliklinik III, University Hospital Carl Gustav Carus Dresden, 01307 Dresden, Germany; 9 Institute of Clinical Chemistry and Laboratory Medicine, University Hospital Carl Gustav Carus, Medical Faculty Carl Gustav Carus, Technische Universität Dresden, 01307 Dresden, Germany; 10 Eunice Kennedy Shriver National Institute of Child Health and Human Development, NIH, Bethesda, MD 20892, USA; 11 Green Templeton College, University of Oxford, Oxford OX2 6HG, UK; 12 NET Unit, ENETS Centre of Excellence, Royal Free Hospital, London NW3 2QG, UK; 13 The LOOP Zurich–Medical Research Center, CH-8091 Zurich, Switzerland

**Keywords:** Paraganglioma, HNPGL, embolization, hypoxia, PD-L1, HIF2α

## Abstract

**Simple Summary:**

In solid tumors, hypoxia activates pathways associated with tumor progression and induces alterations in the immune microenvironment such as the upregulation of programmed cell death-ligand 1 (PD-L1). Pheochromocytomas and paragangliomas (PPGLs) are rare neuroendocrine tumors which are all considered to have metastatic potential independent of their initial clinical presentation. However, the relationship between hypoxia and PD-L1 regulation in PPGLs is still largely unexplored. We show that PD-L1 expression in head and neck paragangliomas (HNPGLs) undergoing embolization (median PD-L1 positivity: 15%) was significantly higher as compared to PD-L1 expression in HNPGLs without prior embolization (median PD-L1 positivity: 0%). Consistently, significantly more HNPGLs with embolization were positive for the hypoxia-marker HIF-2α (median nuclear HIF-2α positivity: 40%) as compared to HNPGLs without embolization (median nuclear HIF-2α positivity: 0%). Our findings suggest, that hypoxia leads to the upregulation of both PD-L1 and HIF-2α in HNPGLs, and may thus facilitate targeted treatment with HIF-2α and checkpoint inhibitors.

**Abstract:**

Hypoxia activates pathways associated with tumor progression, metastatic spread, and alterations in the immune microenvironment leading to an immunosuppressive phenotype. In particular, the upregulation of PD-L1, a target for therapy with checkpoint inhibitors, is well-studied in several tumors. However, the relationship between hypoxia and PD-L1 regulation in pheochromocytomas and paragangliomas (PPGL), and especially in paragangliomas treated with embolization, is still largely unexplored. We investigated the expression of the hypoxia-marker HIF-2α and of PD-L1 in a PPGL-cohort with and without embolization as potential biomarkers that may predict the response to treatment with HIF-2α and checkpoint inhibitors. A total of 29 tumor samples from 25 patients who were operated at a single center were included and analyzed utilizing immunohistochemistry (IHC) for PD-L1 and HIF-2α. Embolization prior to surgery was performed in seven (24%) tumors. PD-L1 expression in tumor cells of head and neck paragangliomas (HNPGLs) receiving prior embolization (median PD-L1 positivity: 15%) was significantly higher as compared to PD-L1 expression in HNPGLs without prior embolization (median PD-L1 positivity: 0%) (*p* = 0.008). Consistently, significantly more HNPGLs with prior embolization were positive for HIF-2α (median nuclear HIF-2α positivity: 40%) as compared to HNPGLs without prior embolization (median nuclear HIF-2α positivity: 0%) (*p* = 0.016). Our results support the hypothesis that embolization with subsequent hypoxia leads to the upregulation of both PD-L1 and HIF-2α in HNPGLs, and could thus facilitate targeted treatment with HIF-2α and checkpoint inhibitors in the case of inoperable, locally advanced, or metastatic disease.

## 1. Introduction

Pheochromocytomas (PHEOs) and paragangliomas (PGLs), together referred to as PPGLs, represent a rare group of endocrine tumors usually associated with catecholamine production [1]. PPGLs have a high degree of hereditability, with germline pathogenic variants (PVs) detected in 30–35% of patients [2,3,4,5]. A further 35–40% of patients with PPGLs harbor somatic PVs [2,3,4,5]. Based on driver genes, PPGLs can be divided into three main molecular clusters: pseudohypoxia cluster 1 (1A and 1B), kinase-signaling cluster 2, and the rare Wnt signaling cluster 3 [2,6,7]. The different clusters are associated with specific biochemical phenotypes, clinical features, and long-term prognosis [6,7,8,9]. 

Tumors belonging to cluster 1 are characterized by pathogenic variants in genes related to hypoxia signaling. As a consequence, hypoxia-inducible factor (HIF)-2α is highly expressed and stabilized in these tumors [10,11] and the transcription of genes related to the hypoxia pathway [10,11] are upregulated. Cluster 1B tumors bear PVs in the von Hippel–Lindau (*VHL*) tumor suppressor gene, HIF-2α encoding gene *HIF2*α/*EPAS1*, or the Egl-9 prolyl hydroxylase-1 and -2 encoding gene (*EGLN 1/2* encoding PHD 1/2) [2,7,9]. PVs in *VHL* result in impaired binding of the VHL protein to HIF-α, which in turn directly affects HIF degradation and consequently stabilizes HIF-α [12]. Increased expression of HIF-2α in PPGLs was shown to be associated with tumor progression and a pro-metastatic phenotype [13,14]. It can be speculated that PPGLs with PVs in pathways involved in HIF-α stabilization under normal oxygen conditions, such as cluster 1A and especially also Custer 1B tumors, might be susceptible to treatment with HIF-2α inhibitors.

Belzutifan (MK-6482, previously known as PT2977) is a selective small molecule inhibitor of HIF-2α [15] and was approved by the FDA in 2021 for the therapy of certain cancers (renal cell carcinoma, pancreatic neuroendocrine tumors, central nervous system hemangioblastoma) associated with von Hippel–Lindau (VHL) disease [16]. In a phase 2 trial (NCT03401788), 22/61 patients had pancreatic neuroendocrine tumors (panNETs) associated with VHL disease [17]: the overall response rate for the subgroup of panNETs was remarkably high at 91% (20/22) [17]. Currently, a phase 2 trial of belzutifan in advanced PPGLs and panNETs is recruiting (NCT04924075) [18]. A newly developed HIF-2α inhibitor, DFF332, is in a phase 1/1b trial tested as a single agent or in combination with everolimus (mTOR inhibitor), but also in combination with spartalizumab (anti-PD1 antibody, see below) plus taminadenant (an adenosine A2A receptor antagonist) (NCT04895748) in patients with advanced clear cell renal cell carcinoma and other malignancies with HIF-stabilizing PVs. Patients with metastatic PPGLs and cluster 1 PV are also eligible to participate in this trial. 

Programmed cell death 1 (PD-1) is expressed on activated T cells and binds to its specific ligand PD-L1 or PD-L2, mainly expressed by antigen-presenting cells [19]. Additionally, pro-inflammatory cytokines can induce the expression of PD-L1 in non-hematopoietic tissue [20]. In conventional T cells, the binding of PD-1 to PD-L1 leads to inhibitory intracellular signaling and induces T cell dysfunction or apoptosis, whereas in T regulatory cells, the differentiation and suppressive function is enhanced by the binding of PD-L1 and PD-1 [19,21]. Cancer cells frequently overexpress PD-L1 as a mechanism to escape from an immune response [19,22].

Programmed cell death-ligand 1 (PD-L1) is a target of the class of checkpoint inhibitors, which block the interaction between programmed cell death-1 (PD1) on T cells and programmed cell death-ligand 1 (PD-L1) on tumor cells, thus preventing the inhibitory signal on T cells and restoring antitumoral T lymphocyte activity [19]. Anti-PD1 and anti-PD-L1 antibodies have shown huge potential and revolutionized the treatment of a wide range of solid tumors such as melanoma, non-small lung carcinoma, Hodgkin lymphoma, and urothelial carcinomas [19].

Intriguingly, the hypoxia-associated pathway upregulates the expression of PD-L1 in malignant cells via HIF-1α and HIF-2α, thus promoting immune tolerance within the tumor microenvironment [23,24,25]. Checkpoint inhibitors have so far been tested in two clinical phase 2 studies in PPGLs [26,27]. In 11 patients treated with pembrolizumab, the disease control rate was 73% at ≥4 months (nine cycles), with a median progression free survival of 5.7 months [26]. In a further study with pembrolizumab, nine metastatic PPGL patients exhibited a disease control rate (DCR) of 75% at ≥4 months and a non-progression rate at 27 weeks of 43%, respectively [26,27]. 

PD-L1 expression on tumor cells—in addition to tumor mutational burden (TMB) and microsatellite instability (MSI) —is a widely used biomarker to predict the response to immunotherapy with antibodies blocking the PD-L1/PD1 axis [28]. However, PD-L1 expression and its association with HIF-α expression in different subgroups of PPGLs is not well studied, and has especially not yet been studied in the context of embolization-induced hypoxia [29,30,31].

Embolization prior to surgery is an established procedure, mainly applied in head and neck paragangliomas (HNPGLs), in order to reduce blood loss from the highly vascular tumors [32,33]. Abdominal PGLs and pheochromocytoma are not embolized prior to surgery on a regular basis. 

In order to better characterize the prevalence of biomarkers potentially predicting the response to targeted treatment with HIF-2α inhibitors and checkpoint inhibitors, we explored the expression pattern of the hypoxia markers HIF-1α and HIF-2α as well as PD-L1, with and without embolization, in our diverse and well-characterized PPGL cohort, and we investigated their possible correlation with clinical, biochemical, and genetic features.

## 2. Materials and Methods

### 2.1. Study Population

In this study, we initially included 19 consecutive patients diagnosed with a PPGL, at the University Hospital Zurich between January 2020 and June 2023, who underwent surgery as a primary treatment in a total of 22 tumors. Inclusion criteria were a diagnosis of a metastatic or non-metastatic PPGL, surgery for resection of the PPGL (*n* = 7 HNPGLs with prior embolization, *n* = 15 PGLs and PHEOs without prior embolization), plus the availability of tumor tissue for immunohistochemical analysis and accessible clinical records. We then enriched our cohort with six additional PPGL patients (with a total of seven tumors) who had undergone resection of an HNPGL between 2010 and 2020 without prior tumor embolization. All patients signed a written informed consent available prior to participation; in the case of children, written parental consent was obtained. Clinical-pathological characteristics were retrieved from medical records. The study was conducted in accordance with the Declaration of Helsinki, and approved by the the Ethics Commission of the Canton of Zurich under the reference number BASEC 2017-00771.

### 2.2. Immunohistochemistry

Altogether, 29 tumor tissue samples from 25 patients were analyzed in the Department of Pathology at the University Hospital Zurich for this study. Immunohistochemistry (IHC) for PD-L1, Ki-67, HIF-2α, and HIF-1α was performed on formalin-fixed, paraffin-embedded representative tumor tissue from resection specimens through a standard immunohistochemical technique, using the Leica Bond III autostainer according to the manufacturer’s protocol. Four-micrometer-thick tissue sections were deparaffinized in dewax solution (Leica, Nussloch, Germany) and hydrated in Bond Wash solution (Leica). Heat-induced epitope retrieval was performed for 30 min (PD-L1 and HIF-1α) and 60 minutes (HIF-2α), respectively, each one at 100 °C in the manufacturer’s solution 2 (Leica). Slides were then incubated with the primary antibodies (PD-L1 clone E1L3N [monoclonal], Cell Signaling Technology, Danvers, Massachusetts, USA, RRID: AB_2687655 and HIF-2α [polyclonal], Abcam limited, Cambridge, United Kingdom, RRID: AB_300582; HIF-1α [monoclonal], Abcam Limited, Cambridge, United Kingdom, RRID AB_302234) at a dilution of 1/100 (PD-L1), 1/50 (HIF-2α) and 1/400 (HIF-1α), respectively, at room temperature. Antibody detection was performed for both stains using the Bond Polymer Refine Detection Kit (DS9800, Leica Biosystem, Wetzlar, Germany) according to the manufacturer’s protocol. Slides were then counterstained with hematoxylin. Adequacy of the reaction was assured by positive controls available for every immunohistochemical stain performed.

The immunohistochemical protocol for Ki-67 has already been described in our previous study [34]. 

Two board-certified pathologists (U.M. and J.F.) independently assessed the immuohistochemistry. One of the two pathologists was blind to the clinical information of the examined samples. The expression of PD-L1 was evaluated counting the percentage of positive tumor cells (positive tumor cells/total tumor cells × 100%). Tumor cells were considered positive if they showed a moderate to strong membranous expression [35]. HIF-2α staining was evaluated as the percentage of tumor cells showing a moderate or strong cytoplasmic and/or nuclear reaction. HIF-1α staining was evaluated as the percentage of tumor cells showing a moderate or strong cytoplasmic and/or nuclear reaction. Ki-67 expression in the 29 tumor samples was evaluated counting the number of tumor cells with a positive nuclear reaction of any intensity in hotpots of at least 100 tumor cells (positive tumor cells/tumor ce;lls × 100%).

### 2.3. Statistics

The Kruskal–Wallis test, Pearson Chi-square-test, and Kendall’s tau test, where appropriate, were used to compare histopathological characteristics between groups of patients. A *p*-value of <0.05 was used as a threshold for statistical significance. Given the low number of cases of this study and the exploratory nature of statistical testing, no adjustment for multiple testing was carried out. Statistical analyses were performed using SPSS Version 29.0.1.1 (IBM Corp. Armonk, NY, USA, Released 2022).

## 3. Results

### 3.1. Patient Characteristics

In this study, 10 patients with PHEOs, 15 patients with extra-adrenal PGLs, and 12 patients with at least one HNPGL were included. In two patients, tissue from two HNPGLs, each resected at different time points, was available. From a further two patients, one HNPGL and one mediastinal PGL were included. Patient characteristics are summarized in Table 1. Of the 25 patients, 13 were female (52.0%). Sequencing data (germline or somatic or both) were available in 22/25 (88%) of patients. Pathogenic variants belonging to cluster 1A were detected in 8/25 (32%) of patients. Pathogenic variants belonging to cluster 2 were present in 7/25 (28.0%) patients. Embolization 1-5 days prior to surgery was performed for 7/29 (24%) of tumors. None of the patients developed metastatic disease during the follow-up period, nor was there any tumor recurrence at the same location after preoperative embolization and surgical removal. 

### 3.2. PD-L1 Expression

**Figure 1:**
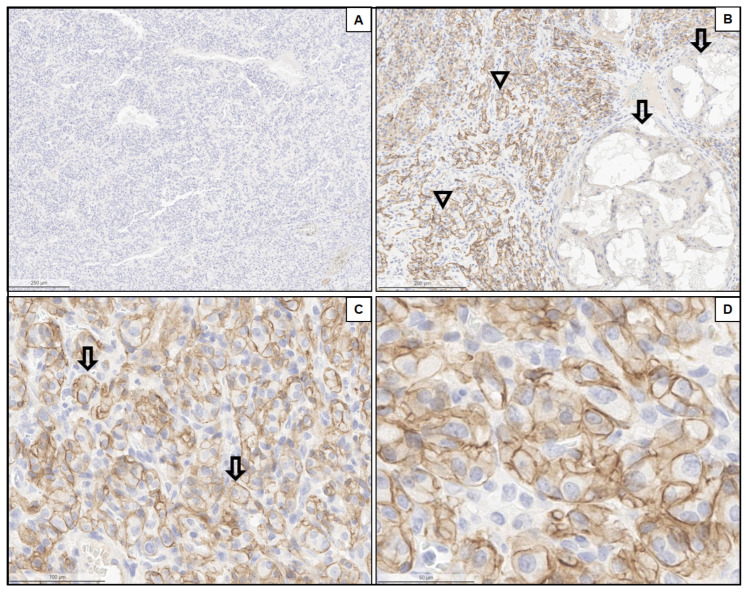
Representative immunohistochemical stains for PD-L1 (Clone E1L3N). (**A**) Non-embolized HNPGL showing no expression of PD-L1 in the tumor cells (magnification: 2.5×). (**B**) Embolized HNPGL demonstrating membranous staining for PD-L1 in the tumor cells. Arrowheads: membranous expression in tumor cells; arrows: obstructed vessels with embolization material (magnification: 5×). (**C**,**D**) Details of previous images showing PD-L1 expression (arrows) at greater magnifications (magnification: 20× [**C**]; magnification: 40× [**D**]).

PD-L1 staining was available for all 29 tumor samples. PD-L1 expression in the tumor cells ranged from 0% to 60% (median 0%). The correlation between the evaluation of the two pathologists was excellent (*Pearson* correlation coefficient *r* = 0.96). Representative images of PD-L1 expression are shown in Figure 1.

In PHEOs, the expression of PD-L1 in the tumor cells was negative (median expression 0%). In abdominal and thoracic PGLs that did not undergo embolization, PD-L1 expression was also negative (median expression 0%) in all cases.

**Figure 2:**
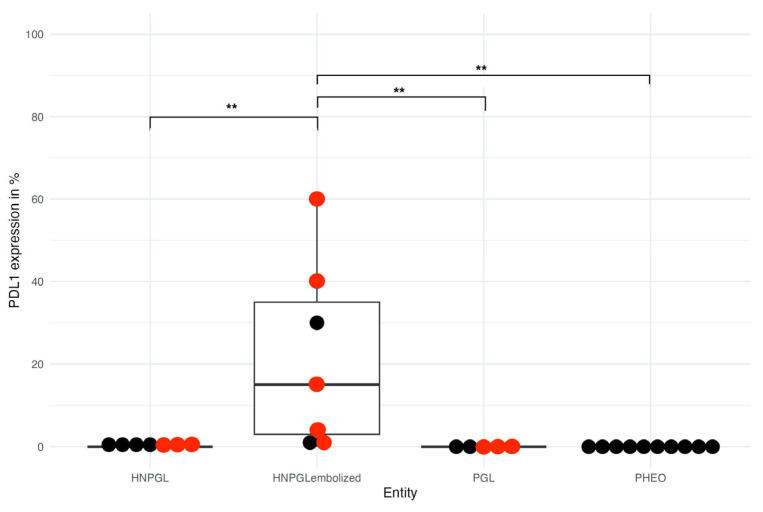
Percentage of tumor cells with PD-L1 expession in different subgroups of PPGLs. Tumors with pathogenic variants in cluster 1A are represented with red dots. Non-cluster 1A tumors are represented by black dots. ** *p* < 0.01. Abbreviations: PHEO: pheochromocytoma; PGL: paraganglioma; HNPGL: head and neck paraganglioma.

In HNPGLs treated with embolization prior to surgery (*n* = 7), the median expression of PD-L1 in tumor cells was significantly higher with 15% (range 0% to 60%) as compared to a median PD-L1 expression of 0% (range 0% to 1%) in HNPGLs without embolization prior to surgery (*n* = 7; *p* = 0.008) (Figure 2). Interestingly, in patient #6 (*SDHB* germline pathogenic variant), the cervical HNPGL was not embolized prior to resection and did not express PD-L1. In contrast, the submandibular HNPGL from the same patient which was resected six months later was embolized prior to surgery, and a PD-L1 expression of 15% was observed in tumor cells. Similarly, in patient #12 (*SDHD* germline pathogenic variant), the para-aortal abdominal PGL resected without prior embolization was negative for PD-L1 expression, whereas the HNPGL was embolized and showed a PD-L1 expression of 40% in tumor cells. In patient #23 (negative germline genetic testing), the difference was less marked, with no PD-L1 expression in the non-embolized mediastinal PGL compared to a PD-L1 expression of 4% in tumor cells of the HNPGL resected after preoperative embolization.

### 3.3. HIF Expression 

**Figure 3:**
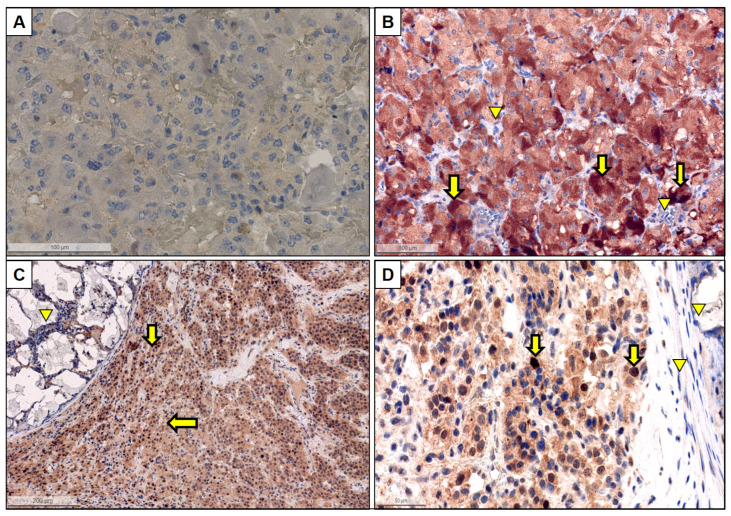
Representative immunohistochemical stains for HIF-2α. (**A**) HNPGL without pre-operative embolization showing no positivity in the tumor cells (neither cytoplasmic nor nuclear, magnification: 20×). (**B**) PHEO showing moderate to strong cytoplasmic expression in the majority of tumor cells (arrows) with adequate negative control (endothelial cells and lymphoid aggregates, arrowheads). Magnification: 10×. (**C**) HNPGL with pre-operative embolization (arrowhead demonstrating embolization material in a vessel) showing diffuse positivity in the tumor cells (arrows). Magnification: 5×. (**D**) Magnification of the previous image showing a diffuse cytoplasmic positivity in the majority tumor cells as well a nuclear positivity in 10–20% of tumor cells (magnification: 20×).

HIF-2α immunohistochemistry was available in 19 tumor samples. There was a very good accordance in the evaluation of HIF-2α expression between the two pathologists (*Pearson* correlation coefficient for number of cells with cytoplasmatic stain *r* = 0.79, *p* < 0.001, *Pearson* correlation coefficient for number of cells with nuclear staining *r* = 0.95, *p* < 0.001). Representative images of HIF-2α staining are provided in Figure 3. One case (patient #18 with *TMEM127* germline PV) was excluded from further analysis due to uninterpretable HIF-2α expression, with diffuse strong positivity in both neoplastic and non-neoplastic cells.

**Figure 4:**
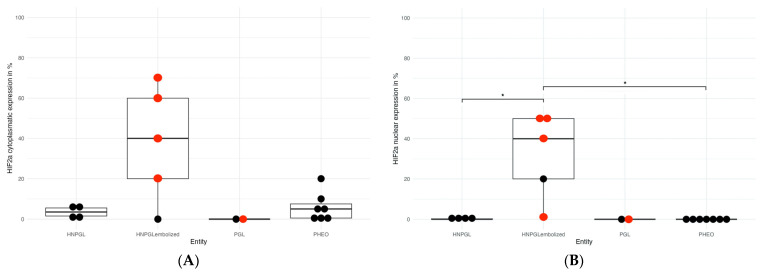
**(A)** Percentage of tumor cells with cytoplasmic HIF-2α nuclear expression in the different subgroups of PPGLs. (**B**) Percentage of tumor cells with nuclear HIF-2α expression in the different subgroups of PPGLs. Tumors with pathogenic variants in cluster 1A are represented with red dots. Non-cluster 1A tumors are represented with black dots. * *p* < 0.05. Abbreviations: PHEO: pheochromocytoma; PGL: paraganglioma; HNPGL: head and neck paraganglioma.

Of 18 evaluable tumors (9 HNPGLs, of these, 5 with and 4 without embolization; 7 PHEOS, of these, 0 with embolization; 2 PGLs, of these, 0 with embolization), 13/18 (72%) were positive for HIF-2α (nuclear or cytoplasmatic staining). All five HNPGLs undergoing embolization prior to surgery (100%) were positive for HIF-2α. Interestingly, four out of five (80%) HNPGLs belonging to this last group showed the nuclear expression of HIF-2α along with cytoplasmic positivity. In detail, cytoplasmic expression was demonstrable in 0 to 40% of tumor cells (median 40%), whereas nuclear expression varied from 1 to 50% (median 40%). In HNPGLs without prior embolization, three out of four tumors (75%) were negative for nuclear HIF-2α expression. In those four HNPGLs without prior embolization, median nuclear expression of HIF-2α was 0% (range 0% to 1%) (Figure 4). Thus, significantly more HNPGLs with prior embolization were positive for nuclear HIF-2α (median nuclear HIF-2α positivity: 40%) as compared to HNPGLs without prior embolization (median nuclear HIF-2α positivity: 0%) (*p* = 0.016) (Figure 4).

In PHEOS, five out of seven (71%) showed cytoplasmic HIF-2α expression (median 5%, range 0% to 20%) whereas nuclear HIF-2α expression was negative in all cases. The two PGLs (abdominal and mediastinal) did not show nuclear or cytoplasmatic HIF-2α expression. 

HIF-1α expression was available in all 29 cases and showed mainly cytoplasmic postivity in tumor cells (median 0%; range 0% to 40%). Limited nuclear positivity (ranging from 2% to 10% of the tumor cells) was demonstrable in six cases, all of which also exhibited cytoplasmic positivity. There was no statistically significant difference of HIF-1α expression (nuclear or cytoplasmic) between the different groups of tumor localization or embolization status.

### 3.4. Association between Immunohistochemistry and Clinical Parameters in Cluster 1A and 2

There was a strong statistical association between PD-L1 expression in the tumor cells and HIF-2α positivity (*Kendall’s tau* correlation coefficient *τb* = 0.864, *p* < 0.001). However, no statistical correlation between PD-L1 expression and HIF-1α or with Ki-67 was detected (Table 1). Ki-67 was also not associated with the expression of HIF-2α or HIF-1α.

Seven patients harbored PVs of one subunit of *SDH* (six with germline PVs, one with somatic PV). Four of them (three germline, one somatic) had at least one HNPGL resected. In this subgroup, HIF-2α IHC and PD-L1 IHC in tumor cells were positive in all four embolized cases. 

**Figure 5:**
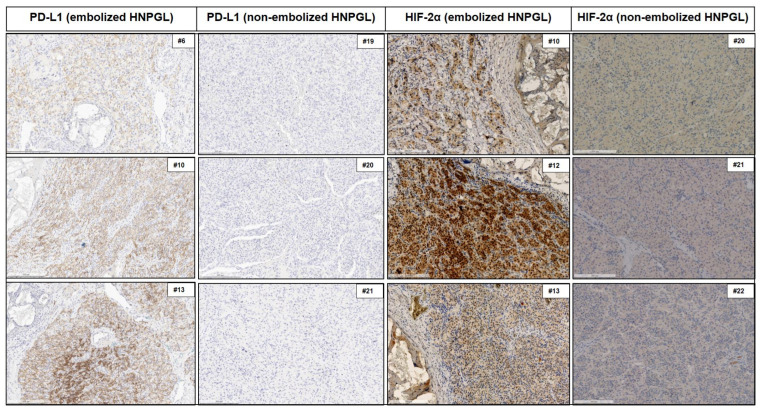
Comparisons between PD-L1 and HIF-2α immunohistochemical staining in embolized vs. non-embolized HNPGLs (for each group three representative cases are shown). The number preceded by hash marks in the upper right box in each figure indicates the patient from whom the illustrated histologic image originated. Magnification: 10×.

Seven tumors harbored PVs in a cluster 2 gene (six PHEOs, one abdominal PGL). HIF-2α was expressed at a very low level in two PHEOs (both *NF1* PVs), and PD-L1 was negative in all cases. There was a statistically significant association of PD-L1 and HIF-2α expression with PVs of cluster 1A tumors compared to cluster 2. However, this association was driven by the fact that, in cluster 2, there were no embolized HNPGLs, whereas in cluster 1A, 5 of 11 tumors were embolized HNPGLs. The results of IHC in patients in which two tumors were available favored additional evidence against the association of cluster 1A with PD-L1 expression. In patients #6 (*SDHB*), #12 (*SDHD*), and #23 (*SDHAF2*), PD-L1 expression was only observed in the embolized HNPGL and not in the non-embolized (HN)PGLs. Representative images of IHC for PD-L1 and HIF-2α in embolized vs. non- embolized HNPGLs are shown in Figure 5.

There was also no correlation between time in hours from embolization to surgical resection in terms of markers of hypoxia (HIF-1α HIF-2α expression) or PD-L1 expression.

## 4. Discussion

In our study, we have shown a significantly higher expression of PD-L1 and nuclear HIF-2α in the tumor cells of HNPGLs that underwent embolization prior to surgical resection, compared to those without embolization. Interestingly, the upregulation of PD-L1 and nuclear HIF-2α in the tumor cells only correlated with embolization-induced hypoxia but not with the pseudo-hypoxia status. 

Endovascular embolization prior to surgery is a long-standing, established procedure in order to reduce blood loss from the highly vascular tumors, and consequently reduce the risk of perioperative complications such as cranial nerve injury [32,33]. As previously reported, angiography after embolization of HNPGLs resulted in an average decrease in blood flow to the tumor by 75% (range 40–95%) [36]. 

### 4.1. Effect of Embolization/Hypoxia on PD-L1 Expression in PPGLs

In our study, 7/29 (24%) PPGLs showed a PD-L1 expression of >1% in the tumor cells, all of these being HNPGLs that were embolized before surgical resection. On the other hand, all the other cases of PGLs (HNPGLs or PGLs from other anatomical locations) that did not undergo embolization before surgery showed no PD-L1 expression, with only one PGL showing expression in 1% of tumor cells. Whether PD-L1 expression is also elevated in pseudo-hypoxia-associated cluster 1 PPGLs remains unclear. In our cohort, PD-L1 and HIF-2α positivity were not associated with cluster 1A PV (pseudo-hypoxia) or cluster 2 PVs when corrected for embolization status. An analysis of 48 PPGL patients in combination with a cohort from “The Cancer Genome Atlas” (TCGA) selected on the presence of matched PV (*n* = 72) even revealed decreased mRNA expression of the *PD-L1* gene in the pseudo-hypoxia cluster 1 compared to the kinase signaling cluster 2 [29]. 

### 4.2. Effect of Embolization/Hypoxia on Hypoxia Markers HIF-1α and HIF-2α Expression in PPGLs

In our cohort, all HNPGLs with prior embolization and available HIF-2α staining were positive for the hypoxia marker HIF-2α (median nuclear HIF-2α positivity: 40%), as compared to HNPGLs without prior embolization (median nuclear HIF-2α positivity: 0%). The expression of HIF-2α has recently been studied by Karakaya et al. in a cohort of 149 PPGLs, demonstrating cytoplasmic expression in a significant percentage of PGLs (69% of PGLs, 92% of HNPGLs and in 33% of PHEOs) [37]. Moreover, while in our study Ki67 did not correlate with PD-L1 or HIF-2α expression, in that study, the expression of HIF-2α was associated with higher Ki67, metastasis at presentation, and worse prognosis. Of note, in their study there was no stratification of HNPGL for preoperative embolization and the majority of tumors were negative for the expression of nuclear HIF-2α [37]. The significance of cytoplasmic versus nuclear expression of HIF-2α still needs to be determined. In our cohort, 80% of patients with HNPGLs who underwent presurgical embolization showed nuclear positivity for HIF-2α. In contrast, only one out of four non-embolized HNPGLs showed nuclear positivity for HIF-2α, and then in a low number of tumor cells. We might suggest that those cases with nuclear positivity for HIF-2α represent tumors with a transcriptionally active HIF-2α, whereas cytoplasmic-only positivity may be an epiphenomenon due to a pseudo-hypoxic pattern, such as in tumors belonging to cluster 1. In our study, HIF-1α did not correlate with hypoxia, nor with tumor location, nor mutational status. In fact, experimental data suggested that HIF-2α rather than HIF-1α is the major transcriptional regulator of a prolonged hypoxic responses [38,39], such that nuclear HIF-2α expression in embolized HNPGLs may be a consequence of hypoxia induced by embolization rather than a mere association with a pseudo-hypoxic phenotype. 

Further studies, and in particular transcriptomic studies, are needed to investigate the significance of these findings and to correlate the transcriptomic data with immunohistochemical results in order to validate the use of immunohistochemical positivity for nuclear HIF-2α as a surrogate marker for transcriptionally active HIF-2α. 

### 4.3. Association of Embolization/Hypoxia, PD-L1 Expression/Immunosuppressive Phenotype and Hypoxia Marker HIF-2α

Four of the five embolized HNPGLs showing nuclear expression of HIF-2α demonstrated a positivity for PD-L1 in >30% of tumor cells, whereas one embolized HNPGL with nuclear expression of HIF-2α showed a PD-L1 expression in 2% of tumor cells. Our results therefore suggest an association between embolization-induced hypoxia, HIF-2α, and PD-L1 expression in HNPGLs. In contrast, Celada et al. found that HIF-2α expression was negatively associated with an immunosuppressive phenotype but positively associated with PVs in the *SDH* gene known to have a pseudo-hypoxic phenotype [31]. However, Celada et al. did not differentiate between nuclear and cytoplasmatic HIF-2α expression.

### 4.4. Hypoxia and Upregulation of PD-L1 in Other Tumor Entities Proteins of the HIF Family

A similar association between hypoxia and PD-L1, as demonstrated in our study, has been described in vivo for cells of the immune system. In a study by Noman et al. (2014), hypoxia not only led to a rapid upregulation of PD-L1 in splenic myeloid-derived suppressor cells in a tumor-bearing mouse model, but also increased the expression of PD-L1 in macrophages, dendritic cells, and tumor cells [40]. In this study, the upregulation of PD-L1 under hypoxic conditions was, in contrast to our study, dependent on HIF-1α but not HIF-2α [40]. Analogously, trans-arterial chemo-embolization (TACE) in hepatocellular carcinoma (HCC) led to a significantly higher PD-L1 expression in HCC tumor cells as compared to HCCs resected without TACE pretreatment (2% versus 0.4%, *P* = 0.027) [41]. In a rat HCC model, intratumoral and peritumoral regions with PD-L1 and HIF-1α IHC positivity were significantly larger after hepatic artery embolization compared to a sham procedure [42]. The association between PD-L1 expression and hypoxia was also documented in a study on 120 glioma patients’ tissues, in which the PD-L1 expression (positive in 42% tumors, mostly overexpressed in high grade gliomas) was significantly related to high HIF-1α expression [43]. In the same study, combination treatment with a HIF-1α inhibitor and anti-PD-L1 antibody in a glioma murine model inhibited tumor growth more profoundly as compared to either monotherapy [43]. Interestingly, PD-L1 expression as evaluated by Western blot and real-time PCR in human muscle-invasive bladder cancer cell lines decreased under hypoxic conditions [44]. Only when cells were grown at the highest cell seeding density was an increase in expression of PD-L1 observed [44]. 

Collectively, these results confirm the association between hypoxia in the tumor microenvironment and PD-L1 expression. However, the detailed crosstalk between the proteins and transcription factors involved are not fully understood. This is underlined by the fact that we found an association of “real” hypoxia with nuclear HIF-2α expression but not with HIF-1α expression.

### 4.5. Limitations of the Study

The main limitation of this study is the small number of patients and tumor samples included. Thus, the strength of our conclusions is limited. However, considering the rarity of this tumor entity, the acquisition of more tumor samples is problematic.

Furthermore, HIF-2α staining was not available for all tumor samples due to the unavailability of reagents for the established assay. No mechanistic studies were performed and we only investigated expression pattern by immunohistochemistry. Nonetheless, our study explores the association between hypoxia due to embolization, PD-L1, HIF-1α, and HIF-2α at immunohistochemical level through reproducible methods that may be employed in routine pathology practice. Moreover, we have examined tumor sections, thus reducing the bias due to tumor heterogeneity that typically affects studies based on tissue microarrays (TMAs). 

## 5. Conclusions

In this study, we show that embolization-induced hypoxia, but not pseudo-hypoxia, led to PD-L1 and nuclear HIF-2α upregulation by IHC in HNPGLs. Further studies are needed to elucidate whether this finding may be associated with a response to checkpoint or HIF-2α inhibitors or the combination of both in inoperable, locally advanced, or metastatic PPGLs. 

## Figures and Tables

**Table 1:** Patient characteristics.

Patient ID	Tumor type and Localization	Sex	Age	Germline Sequencing	Tumor Sequencing	Cluster	Biochemical Phenotype	Ki-67 (in %)	PD-L1 (in %)	HIF-2α Nuclear (in %)	HIF-1α Cytoplasmatic (in %)	Embolization Prior to Surgery
1	PHEO, metastastic	F	65	Negative	*ATRX*	-	Noradrenergic	4	0	0	0	No
2	PGL abdominal, metastatic	M	38	*SDHB*	*SDHB*	1A	Noradrenergic	20	0	0	0	No
3	PHEO	M	76	Negative	*NF1*	2	Noradrenergic	2	0	0	30	No
4	PHEO	F	62	Negative	Negative	-	Noradrenergic	3	0	0	10	No
5	HNPGL	F	27	*SDHB*	*SDHB*	1A	Silent	10	2	40	30	Yes
6	HNPGL (2)	F	47	*SDHB*	*SDHB*	1A	Silent	5 (both)	0, 15 (ebmolized HNPGL)	N/a; 1 (HNPGL embolzed)	0, 0	1 HNPGL yes, 1 HNPGL no
7	PHEO	M	44	Negative	*NF1*	2	Noradrenergic	2	0	0	0	No
8	PHEO	M	42	*NF1*	n/a	2	Adrenergic	2	0	0	0	No
9	PHEO (bifocal)	M	50	*NF1*	n/a	2	Adrenergic	2	0	N/a	0	No
10	HNPGL	F	61	Negative	Negative	-	Dopaminergic	2	30	20	20	Yes
11	PHEO	F	76	Negative	Negative	-	Noradrenergic	0.5	0	N/a	0	No
12	PGL (abdominal and HNPGL)	M	25	*SDHD*	n/a	1A	Noradrenergic	3	0, 40 (HNPGL)	N/a, 50 (embolized HNPGL)	0, 5	Abdominal PGL: no; HNPGL: yes
13	HNPGL	M	54	Negative	*SDHD*	1A	Adrenergic	2	60	50	10	Yes
14	PGL	M	42	Negative	*HRAS*	2	Adrenergic	0	0	0	0	No
15	PHEO	M	51	Negative	negative	-	Adrenergic	0	0	0	0	No
16	PHEO	F	65	Negative	*NF1*	2	Adrenergic	7	0	0	0	No
17	PGL paraaortal	F	30	*SDHC*	n/a	1A	Noradrenergic	4	0	N/a	0	No
18	PHEO	F	29	*TMEM127*	n/a	2	Adrenergic	8	0	N/a	0	No
19	HNPGL	F	47	n/a	n/a	-	N/a	8	0	0	0	No
20	HNPGL	F	26	n/a	n/a	-	N/a	6	0	0	0	No
21	HNPGL	M	32	Negative	n/a	-	Noradrenergic	8	0	1	10	No
22	HNPGL	M	62	n/a	n/a	-	N/a	4	0	0	20	No
23	PGL mediastinal and HNPGL	F	34	Negative	n/a	-	Silent	4-7	0, 4 (HNPGL)	N/a	0, 0	Mediastinal: no; HNPGL: yes
24	HNPGL (2)	M	15	*SDHAF2*	n/a	1A	Silent	10-15	0 (both)	N/a	0, 40	1 HNPGL yes, 1 HNPGL no
25	HNPGL	F	27	*SDHB*	n/a	1A	Silent	7	1	N/a	10	No

Abbreviations: PHEO: pheochromocytoma; PGL: paraganglioma; HNPGL: head and neck paraganglioma; M: male; F: female; VUS: variant of unknown significance; Ki-67: proliferation index estimated through immunohistochemistry; n/a: not available.

## Data Availability

The data presented in this study are available in Table 1.
